# Direct Isolation of Carboxylated Cellulose Nanocrystals from Lignocellulose Source

**DOI:** 10.3390/polym17152124

**Published:** 2025-07-31

**Authors:** Thai Anh Do, Luong Lam Nguyen, Thuy Khue Nguyen Thi, Van Quyen Nguyen

**Affiliations:** 1 Department of Advanced Materials Science and Nanotechnology, University of Science and Technology of Hanoi, Vietnam Academy of Science and Technology, 18 Hoang Quoc Viet, Cau Giay, Hanoi 11307, Vietnam; anhdt.m22amsn@usth.edu.vn (T.A.D.); nguyen-luong.lam@usth.edu.vn (L.L.N.); 2 Faculty of Pharmacy, Haiphong University of Medicine and Pharmacy, 72A Nguyen Binh Khiem, Ngo Quyen, Ha Phong 180000, Vietnam; nttkhue@hpmu.edu.vn

**Keywords:** lignocellulosic source, carboxylated cellulose nanocrystals, dragon fruit foliage, peracetic acid

## Abstract

In this study, we report an effective, one-step chemical treatment to directly isolate carboxylated cellulose nanocrystals (CCNCs) from a lignocellulosic source using a mixture of peracetic acid and 10% H_2_SO_4_ solution. We used infrared spectroscopy, X-ray diffraction, dynamic light scattering, atomic force microscopy, and scanning electron microscopy to characterize all the materials. The obtained CCNCs exhibited needle-like shapes with a width of 10–50 nm and a length of 200–500 nm, a high crystalline index (71.3%), and a high content of -COOH groups (~1.405 mmol/g), with a zeta potential value of −48.5 mV. We attributed this to the cooperative effect of strong oxidative agent and strong acid, which makes the removal of all components occur simultaneously in parallel with the partial hydrolysis of amorphous cellulose regions. Our study opens a new, simple approach to directly isolate cellulose nanocrystals from a lignocellulosic source.

## 1. Introduction

Cellulose nanomaterials have recently emerged as one of the most promising materials thanks to their abundance, regeneration in the biosphere, and unique physico-chemical properties, including high mechanical strength, reinforcing capabilities, natural degradability, environmental friendliness, and, especially, biocompatibility [1,2,3,4,5]. Cellulose nanomaterials show great potential in a wide range of applications, for example, as a reinforcing agent in biodegradable plastics, as wound dressings [6,7], as antimicrobial [8,9,10,11,12,13,14,15,16,17,18] and antibacterial gels [19], as tissue engineering scaffolds in biomedical fields [20], as bio-adsorbents in soil and water treatment [21], or as ionic conductors in energy storage and conversion [22,23,24,25,26,27]. For example, Kono et al. reported that cellulose nanofibers modified with quaternary ammonium salts are antimicrobial against *Klebsiella pneumoniae*, a Gram-negative bacterium, and attributed this to the positively charged surface of the modified cellulose [28]. Gutierrez et al. reported that cellulose-based hydrogels with in situ-synthesized copper exhibited antimicrobial behavior against *E. coli* and *S. aureus* strains [8]. Valencia et al. reported that the carboxylated cellulose nanofibrils can spontaneously grow Cu_2_O nanoparticles, and the new hybrid Cu_2_O-CNCs are antimicrobial against both Gram-negative (*E. coli*) and Gram-positive (*L. innocua*) bacteria [29].

Cellulose nanomaterials were first produced by Ränby in a form of a colloidal suspension after hydrolysis of cellulose in concentrated H_2_SO_4_ [30]. Transmission electron microscopy revealed needle-shaped nanoparticles, and electron diffraction later confirmed the crystalline cellulose I structure of the nanoparticles [30]. In terms of dimensions, cellulose nanomaterials can be classified into (i) cellulose nanocrystals (CNCs) with a width of few 10 nm and length of few 100 nm and (ii) cellulose nanofibers with a width of few 10 nm and length of few μm [30,31,32]. It is known that the exact size of cellulose nanomaterials also depends on the preparation method and the lignocellulosic source [32,33,34].

**Scheme 1:**
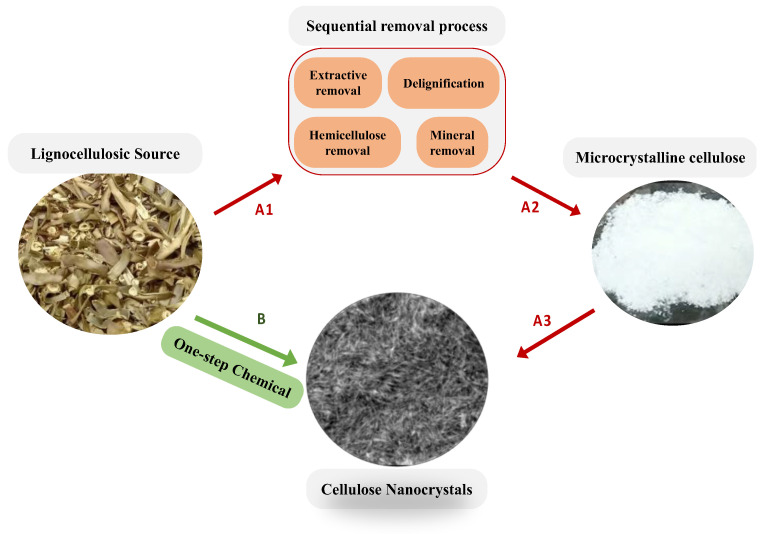
Schematic presents (**A**) general procedures to extract cellulose nanocrystals (CNCs) from lignocellulosic source with red arrow (A1, A2, and A3) showing the sequential process. (**B**) Our one-step chemical treatment to directly extract CNCs using a mixture of peracetic acid with 10% H_2_SO_4_.

Generally, cellulose nanomaterials can be extracted from lignocellulose sources by several treatment processes, illustrated as red arrows (A1, A2, and A3) in Scheme 1. At first, a lignocellulosic source was collected, pretreated with water, and then air-dried. Afterward, the dried lignocellulosic source is milled into particles of μm size and stored for further use. The second step (as presented in the sequential removal process box in Scheme 1) was to remove wax, extractives, lignin, minerals, and hemicellulose to extract microcrystalline cellulose by chemical treatment with or without mechanical treatment [33,34,35,36,37,38]. It is known that the lignocellulosic biomass usually consists of a defensive inner structure which contributes to the hydrolytic stability and structural robustness of the plant cell walls, and, in addition, the existence of crosslinks between cellulose and hemicellulose with lignin via ester and ether linkages leads to the biomass’s recalcitrance [39]. As a result, the delignification and purification process strongly depends on the lignocellulosic source and must usually be repeated several times to obtain the MCC, causing serious problems, such as time and energy consumption, equipment corrosion, and severe environmental pollution. The final step of cellulose nanomaterial extraction, as illustrated by the arrow A3 in Scheme 1, is to hydrolyze the amorphous regions to obtain cellulose nanocrystals (CNCs) or to oxidize the -CH_2_OH group to extract cellulose nanofibers [32,40]. As a result, it is highly desirable that the lignocellulosic source can be directly converted into nanocrystal cellulose by a one-step chemical treatment, as illustrated by the green arrow B in Scheme 1. Yang et al. reported that carboxylate cellulose nanocrystals can be directly extracted from the borer powder of bamboo using ammonium persulfate [41], and a few others also reported the one-step approach to prepare CNCs or CCNCs [42,43].

Here, we report a direct isolation of carboxylated cellulose nanocrystals (CCNCs) from lignocellulosic sources, particularly the cores of dragon fruit foliage (WC-DFF), by using a mixture of peracetic acid (PAA) with 10% H_2_SO_4_. The peracetic acid added with 10% H_2_SO_4_ (PAA-10H_2_SO_4_) is of interest for two reasons: First, the peracetic acid, as an emerging oxidant and disinfectant, has increasingly been used in wastewater treatment, food, and medical industries, making PAA a promising candidate as a green oxidizing solvent [44,45]. Second, we have proven that peracetic acid with 0.5 to 5% (*w*/*w*) H_2_SO_4_ can bleach lignocellulosic source in one-step treatment at 80 °C in 2 h to obtain microcrystalline cellulose (with a yield of 47.5% for DFF) and part of the result was shown in the current report. Based on these findings, we asked a question “what if we increased the H_2_SO_4_ concentration in the PAA solution up to 10% and is it possible to directly isolate cellulose nanocrystals?”. The obtained CCNCs have needled-like shapes with a width of 10–50 nm and a length of 200–500 nm, and a high crystalline index (71.3%), and a high content of -COOH groups (~1.405 mmol/g) with a zeta potential value of −48.5 mV. Our method opens a new path to directly isolate cellulose nanocrystals from lignocellulosic raw materials.

## 2. Materials and Methods

### 2.1. Chemicals

Fine-dried Dragon Fruit foliage (DFF) was obtained by mechanically milling air-dried samples, it was then stored in a sealed container. Analytical grade chemicals including hydrogen peroxide (30% *w*/*w* in water), acetic acid (>99.7%), and sulfuric acid (98%) were purchased from Sigma-Aldrich and used without further purification.

Bleaching lignocellulosic source by PPA-2H_2_SO_4_: The PPA-2H_2_SO_4_ solution was prepared by mixing hydrogen peroxide (30%) and acetic acid glacial in equal volumes (20/20 mL), then sulfuric acid 98% (~0.45 mL) was added to the above solution. The biomass was then added to the solution with the liquor ratio of 1:10 (g/mL). The reaction was then conducted at a temperature of 80 °C degrees for a duration of 2 h. The reaction was then terminated by adding cold deionized water, and the mixture was washed and filtered by distilled water until neutralization.

Isolation of nanocrystal cellulose by PPA-10H_2_SO_4_: The solution of hydrogen peroxide (30%) and acetic acid glacial in equal volumes (20/20 mL) was cooled down to 0 degrees Celsius, and then 10% (*w*/*w*) sulfuric acid 98% (~0.9 mL) was slowly added. After that, the solution was kept at an ambient temperature for 1 h before use. The biomass was then added to the mixture with a biomass-to-liquor ratio of 1:10 (g/mL), and the reaction was then conducted at a temperature of 80 °C for a duration of 4 h. The reaction was then terminated by adding cold deionized water, followed by centrifugation at 10,000× *g* rpm for 10 min. The washing process was performed several times until the pH was reached ~7.

### 2.2. Chemical Composition Analysis

To determine the chemical content of raw biomass, bleached samples, and the nanocrystal cellulose samples, we used the protocol published by national renewable energy laboratory with some modifications [46,47]. The measurement was performed at least 3 times for each sample. The following procedure was conducted, in particular, for extractive content: 0.3 g of biomass sample was weighed and placed in a beaker. Then, 30 mL of ethanol was added to the beaker, and the mixture was subjected to an oil bath at 70 °C for a duration of 3 h. Subsequently, the solid residue was filtered and washed with hot ethanol ten times, using 2 mL of ethanol each time. The residue was then dried until a constant weight was achieved and cooled to room temperature. The extractive content was calculated as the weight loss observed after the treatment.

*For lignin content*: An extractive-free sample weighing 0.3 g was placed in a beaker. Next, 3 mL of 72% sulfuric acid was added to the beaker, and the mixture was kept in a water bath at 30 °C for 1 h. After the hour elapsed, 84 mL of distilled water was added to the beaker. The mixture was then subjected to autoclaving at 120 °C for 1 h. Subsequently, the solid residue was filtered and washed with hot distilled water to remove any excess acid. The acid-insoluble residue was dried at 105 °C until a constant weight was achieved, cooled in a desiccator to room temperature, and weighed as m_1_. The residue was then placed into a muffle furnace at 575 °C until a constant weight was obtained. After cooling in a desiccator to room temperature, the weight of the resulting ash was recorded as m_2_. The acid-insoluble lignin content was calculated by subtracting m_2_ from m_1_. The amount of acid-soluble lignin was determined using UV-Vis Spectroscopy.

*For hemicellulose content*: An amount of 0.3 g of extractive-free sample is added to 3 mL of 0.5 M NaOH in a beaker placed in an oil bath at 80 °C for 3.5 h. After that, the residue is washed with distilled water to neutral pH, and then it is dried to a constant weight and cooled to room temperature. The amount of hemicellulose content is the weight loss after the treatment.

*For ash content*: An amount of 0.1 g of biomass sample is added into a pre-weighed crucible, the sample is ignited until no more smoke or flame appears; this is followed by dry oxidation in the 575 °C muffle furnace for 5–6 h. The crucible and sample are then cooled to room temperature in a desiccator. Record the weight of ash content.

### 2.3. Materials Characterization

*Fourier transform infrared spectroscopy:* The chemical structure of the samples was determined by Fourier transform infrared (FT/IR 4700, JASCO, Tokyo, Japan) with attenuated total reflectance (ATR) mode at a wavenumber range of 400 to 4000 cm^−1^ with a step of 2 cm^−1^ and for 32 scans.

*Field Emission Scanning Electron Microscopy (FE-SEM).* The microcrystalline and nanocrystal cellulose solution was spin coated on SiO_2_ (100) wafer, and then the wafer sample was dried at 40 °C under vacuum overnight. The sample was then coated with 15 nm of Ti by e-beam evaporator (lesker PVD-75, Kurt J. Lesker Company, Jefferson Hills, PA, USA) at vacuum 10^−6^ torr and depositing rate 0.2 nm∙s^−1^. The sample was then imaged by FE-SEM (JEOL, JSM-IT800, JEOL, Tokyo, Japan) at few kV. EDS mode was conditioned by using an accelerating voltage of 20 kV, a working distance of 10 mm, and the accumulation time of 1 min.

*Atomic Force Microscopy (AFM).* The AFM was performed by the Asylum research MFP-3D origin, at an ambient temperature in a contact mode with a scan rate of 1 Hz using a silicon nitride tip (Bruker DNP-10, Bruker, Billerica, MA, USA). AFM images were taken in several size scans applying a soft force (~1 nN) on the AFP tip, and then all data were analyzed by the Gwyddion 2.59 software program.

*Particle size and zeta potential:* The particle size distribution and zeta potential of each sample were analyzed by a Nano-ZS analyzer (Malvern Instrument, Worcestershire, UK). CN suspensions (0.05 wt%) were homogenized for 10 min using Vibra Cell MC VCX750 Ultrasonic Processors (Sonics & Materials, Inc., Newtown, CT, USA) at 325 W (ON/OFF, 30 s/15 s). Each sample was measured 3 times, and the value of the zeta potential was estimated on average.

*X-ray Diffraction:* The crystallinity of each sample was determined by X-ray diffraction (D8- ADVANCE X-ray powder diffractometer, Bruker, Ettlingen, Germany), operating at 40 kV voltage and 30 mA using a Ni-filtered Cu K radiation (λ = 0.15406 nm). The sample was scanned at the ambient condition over scattering 2θ from 10° to 70° with the scanning rate of 0.03°/0.7 s. The crystallinity index (*CrI*) was calculated using Segal’s method [48] using the following formula:

(1) $$CrI=\frac{I_{200}-I_{AM}}{I_{200}}\times 100\%$$

where *I*_200_ is the peak intensity at the plane (200) characterized for the crystallinity domain peaked at 2θ~22.8°, while *I_AM_* is the peak intensity at the minimum between (200) and (101) plane peaked at 2θ~18° characterized for the amorphous domain in cellulose.

Thermogravimetric analysis (TGA, Setaram, Cranbury, NJ, USA) was used to analyze the thermal stability and degradation behavior of raw and chemical treated sample. The analysis was performed between ambient and 900 °C under inner gas, with a flow rate of 2.5 L/min at a heating rate of 10 °C/min. The measurement is conducted at the Institute of Chemistry, Vietnam Academy of Science and Technology.

## 3. Results and Discussion

**Figure 1:**
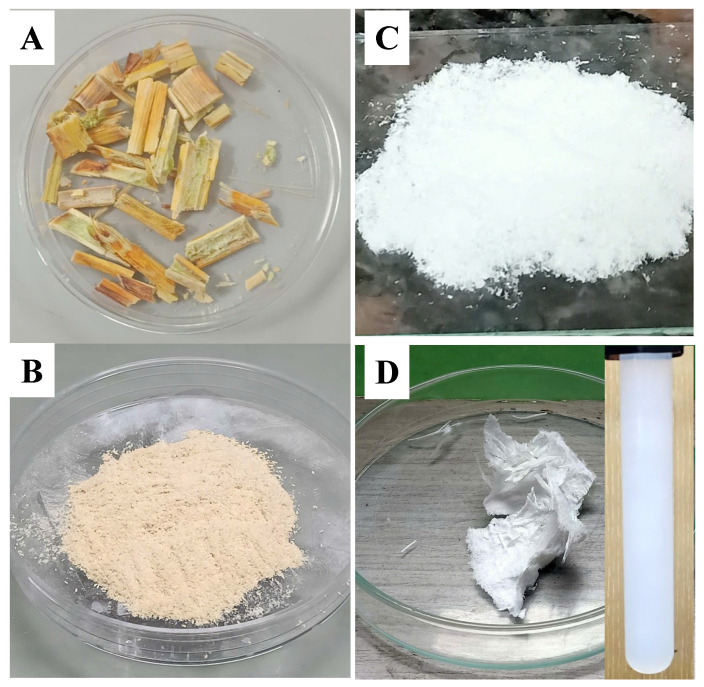
(**A**) Image of the raw woody cores of dragon fruit foliage (WC-DFF), (**B**) image of the powder of the sample (WC-DFF) after grinded, and (**C**) Microcrystalline cellulose (MCC) obtained directly from the reaction between the WC-DFF powder and the PAA-2H_2_SO_4_. (**D**) The lyophilized sample of the carboxylated cellulose nanocrystals (CCNCs) obtained directly from the reaction between the WC-DFF powder and the PAA-10%H_2_SO_4_, with the inset showing the CCNC dispersion (4% w) in water.

Firstly, the woody cores of dragon fruit foliage (WC-DFF) were collected from local fields and then washed several times by distilled water, and they were then oven-dried at 50 °C until the mass unchanged, as shown in Figure 1A. After that, the WC-DFF was ground and filtered through a 60-mesh filter screen to obtain the powder as shown in Figure 1B.

Then, the reaction between the WC-DFF and PAA-2H_2_SO_4_ was performed at a temperature of 80 °C for 2 h. After terminating the reaction, the bleached sample appeared white and had a yield of 47%, as shown in Figure 1C. The color transition indicates the removal of chromophore group in the lignin component, which are mainly responsible for the brownish color and light adsorption properties of natural lignocellulosic sources. Next, we prepared the PAA with 10% of concentrated H_2_SO_4_ and conducted the reaction at 80 °C with the liquor-to-biomass ratio (10:1 mL/g). After 4 h, the cooled distilled water was added to quench the hydrolysis reaction and then centrifugated at 10,000× *g* rpm for 5 min. The washing process was repeated several times until the pH of the solution reached closer to 7. The partially transparent sample was subjected to a dialysis step against deionized water until reaching a neutral pH. Figure 1D shows the image of the sample after the freeze-drying technique, which also indicates the removal of chromophore group in the lignin component. The yield of the reaction was estimated by comparing the weight before and after the reaction, and the obtained yield was 10%, which is much lower than the yield of microcrystalline cellulose (the bleached sample) obtained from the reaction of the WC-DFF and PAA-2H_2_SO_4_. The inset in Figure 1D showed the image of the 4% (w/mL) CCNC redispersion in deionized water after 3 months, with the bluish white color indicating that the colloidal nature of the CCNCs is due to the Tyndall effect.

**Figure 2:**
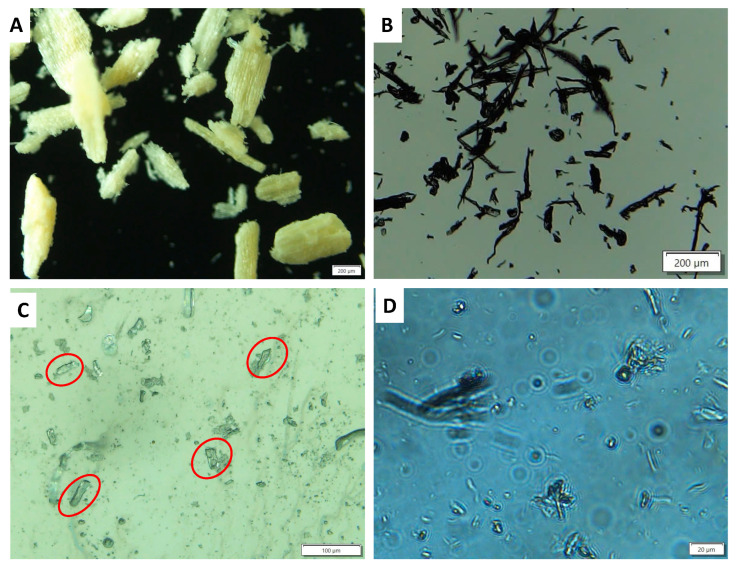
Optical image of (**A**) raw DFF sample (scale bar 200 μm) and (**B**) the bleached sample obtained from the reaction of the WC-DFF with PAA-2H_2_SO_4_ (scale bar 200 μm). (**C**) The sample obtained from the reaction of the WC-DFF with PAA-10H_2_SO_4_ after 2 h (Scale bar 100 μm) and (**D**) after 4 h reaction (scale bar 20 μm).

Interestingly, the chemical reaction including both the bleaching of the raw material and the hydrolysis of the microcrystalline cellulose into nanocrystalline cellulose was complex and made it challenging to precisely monitor the reaction. In the current study, the reaction was initially monitored by following the sample shape and size measured by the optical microscopy (Figure 2). Optical microscopy allows us to check the size of the sample rapidly, and more importantly, the shape of the sample during the reaction. It is noted here that due to the aggregation of nanocrystal cellulose, monitoring the shape of the sample by the optical microscopy is especially necessary to track the reaction. In our experiment, the reaction was divided into two stages. The first stage involves the color change in the lignocellulosic source—from yellow or brown color (depending on the lignocellulosic source) into white—color indication of forming microcrystalline cellulose. The second phase corresponds to the hydrolysis of the biomass into nanocrystal cellulose. Between both phases, there is an intermediate phase where both nanocrystal cellulose and microcrystalline cellulose co-exist.

Figure 2A presents the image of the raw sample, while Figure 2B presents the image of the WC-DFF treated with PAA-2H_2_SO_4_ for 2 h displaying the rectangular shape with the length of 200 to 500 μm, which is later assigned to the microcrystalline cellulose (MCC). Figure 2C illustrates the image of the sample reacted with PAA-10H_2_SO_4_ for 2 h, in which the rectangular shape still appeared, as a red circle, suggesting the intermediate phase where both microcrystalline cellulose and nanocrystalline cellulose co-existed. Figure 2D shows an image of the sample reacted with PAA-10H_2_SO_4_ for 4 h where the microcrystalline cellulose disappeared, suggesting that the hydrolysis of the microcrystal cellulose was complete.

The chemical components of raw material, the bleached WC-DFF or MCC, and the CCNCs were analyzed according to standardized procedures published by national renewable energy laboratory (NREL/TP-510-42618, NREL/TP-510-42619, NREL/TP-510-42621, NREL/TP-510-42622, and NREL/TP-510-48087) and compared it in Table 1. The analysis reveals that the lignin, wax, extractive, and mineral contents of the bleached MCC and CCNCs were significantly reduced, while the cellulose content was significantly increased, which is an indication of the hydrolysis of hemicellulose. It is noted here that the content of hemicellulose of the bleached MCC is 1.5 times greater than that of the CCNCs.

**Figure 3:**
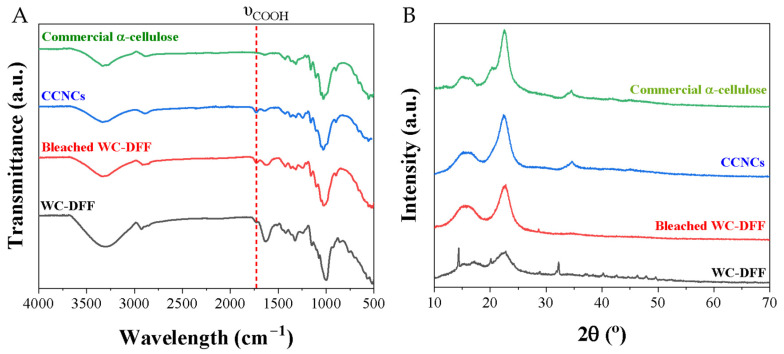
(**A**) FT-IR spectra and (**B**) XRD spectra of the raw WC-DFF, bleached WC-DFF, and CCNC samples in comparison with commercial microcrystalline cellulose.

Figure 3A presents the FTIR spectra of the CCNC samples, the raw samples, commercial microcrystalline cellulose, and the bleached MCC. As shown, our CCNCs show a similar characteristic in comparison with the commercial microcrystalline cellulose and the bleached WC-DFF or MCC sample. There are vibrational bands between 899 and 1163 cm^−1^, which are usually attributed to β-(1-4) glycosidic ether links, and the β-glycosidic linkages, C-O-C glycosidic stretching, C-OH stretching vibration, and C-O stretching, indicating the presence of cellulose structure. These bands at 1426 and 1315 cm^−1^ can be assigned to -CH_2_- symmetric bending groups of cellulose [49]. Importantly, there is a new peak at 1732 cm^−1^ which is attributed to the -COOH functional group [38,50,51]. The content of the -COOH group in our CNCs was quantified by an electrical conductivity method, as mentioned in Section S1. The obtained -COOH content is 1.405 mmol/g with the Zeta potential value of −48.5 mV, which is close to the -COOH content of cellulose nanofibril prepared by TEMPO oxidative agent [52,53,54].

Figure 3B shows the XRD spectra obtained for CCNCs, the bleached WC-DFF, commercial microcrystalline cellulose, and the raw material. The spectra indicated that after chemical treatment with PAA-10H_2_SO_4_ and PAA-2H_2_SO_4_, all mineral impurity in the raw material was removed, evidenced by the disappearance of a broad peak at a large 2θ angle (>30°), which is consistent with EDS result. XRD spectra show characteristic peaks at 15.49° and 22.44° in both the raw and treated sample assigned to the (101) and (002) planes, respectively, and importantly for the CCNC sample there is a new peak at 34.3°, which is a characteristic of the (040) crystal plane [49,55]. Crystallinity indices (*CrIs*) were increased from 43.31% in the raw DFF to 61.30% in the bleached WC-DFF and to 71.30% in CCNC samples, which is smaller than the value of the CNCs obtained via the hydrolysis of microcrystalline cellulose with concentrated H_2_SO_4_, but higher than the value of cellulose nanofibers [49].

**Figure 4:**
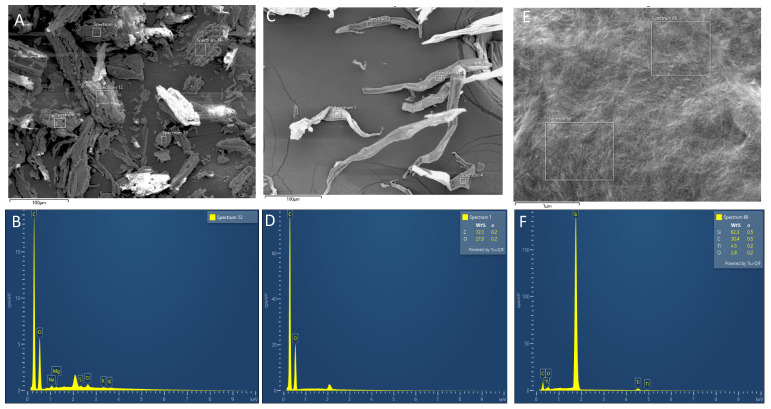
FE-SEM image and its corresponding EDS of (**A**,**B**) the raw WC-DFF, of (**C**,**D**) the bleached WC-DFF, and of the CCNCs (**E**,**F**). The table inset showed the element ratio.

In Figure 4, elemental analysis by EDS of the bleached WC-DFF and CCNCs exhibited only C and O atoms with the atomic ratio close to that obtained from commercial microcrystalline cellulose. It is important to note that the presence of Ti and Si elements in the CCNC sample originated from 20 nm of Ti deposition on top of our CCNCs for SEM observation and Si (100) wafer. The results prove that the chemical treatment was highly effective in removing mineral components.

Figure S1 presents the thermal analysis of CCNCs in comparison with the raw sample. As expected for lignocellulosic material, the decomposition of the CCNCs occurred in three main steps. First, below 200 °C, there is a small weight loss, approximately 10%, which can be assigned to the moisture evaporation. Above 200 °C, there was a larger change in the raw sample compared to the CCNCs, which could be caused by decomposition of the extractive component. From 200 to 450 °C, there is a great weight loss (~75%) caused by cellulose decomposition and tar formation. During the last stage above 450 °C, the cellulosic crystalline structure was completely decomposed into volatiles and tar. It is suggested that the CCNCs and the bleached WC-DFF are almost decomposed with the tar of less than 5% CCNCs, whereas the residual content of the raw sample remained ~10%. The XRD, EDS, and thermal analysis show the significant increase in crystalline and the high removal of mineral, lignin, and hemicellulose content.

**Figure 5:**
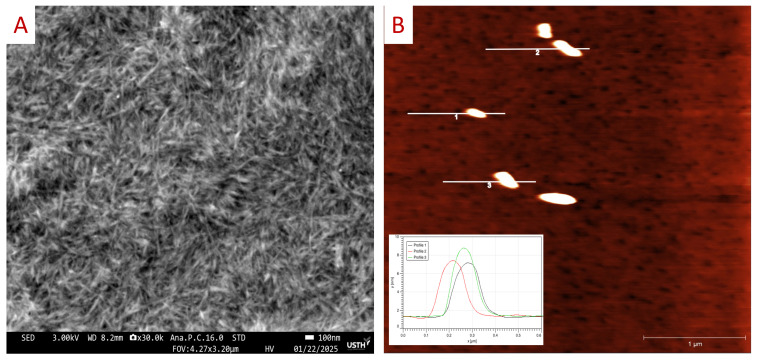
(**A**) SEM image of the CCNC sample deposited on SiO_2_ (100) substrate coated with 20 nm of Ti layer. (**B**) AFM topography of the CCNC sample deposited on mica substrate obtained by contact mode, with the inset showing the height profile of the CCNC particles 1, 2, and 3.

Next, the size of our CCNCs was investigated by atomic force microscopy (AFM) and field emission scanning electron microscopy (FE-SEM). For the FE-SEM observation, we deposited the CCNC suspension (~0.1% wt) on the fresh SiO_2_ substrate (2 × 2 cm^2^), and then a 20 nm Ti layer as the conducting layer was deposited on top of the sample by e-beam evaporator to increase the conductivity of the CNC layer. Figure 5A presents the SEM image of the CCNCs in needle-like shape in which the length of the CNCs was estimated in range of 200–500 nm and a width of 10 to 50 nm. For AFM measurement, a droplet of diluted CCNC suspensions (~0.01% wt) was deposited on a fresh micro substrate (2 × 2 cm^2^), and then dried at ambient temperature. Figure 5B presents the topography of CCNCs (with the height profile of particles 1, 2, and 3 in the inset) obtained by the contact mode, which has the rod-shaped nanoparticle with a width of 20 to 50 nm, length of 300 to 500 nm, and a height of ~10 nm. Figure S2 shows the particle distribution of the CCNCs with the diameter of 190 ± 30 nm obtained from dynamic light scattering measurement. Our dimension analysis confirmed that CCNCs are needled-like shapes with a width of approximately 10 to 50 nm, and length of few 100 nm.

At this stage, it can be concluded that the PAA-10H_2_SO_4_ solution can be used to directly extract carboxylated cellulose nanocrystals in one step. The obtained CCNCs have a high crystalline index (~70%), are rod-shaped with an aspect ratio of 2 to 10, and are functionalized with the -COOH group content (1.405 mmol/g), which results in a zeta potential value of −48.5 mV. To the best of our knowledge, this is the first report proving that it is possible to extract cellulose nanocrystal directly from the lignocellulosic source.

Next, we want to generalize our method and determine the effect of H_2_SO_4_ concentration on CCNC extraction. First, the same experiments were performed for cob corn and banana pseudostem and the obtained CCNCs had a width of approximately 20 nm and length of 300–500 nm, as shown in Figure S3. Second, we performed the hydrolysis of microcrystalline cellulose with PAA-2H_2_SO_4_, 10% H_2_SO_4_, and PAA-10H_2_SO_4_. After the reaction, neither hydrolysis with 10% H_2_SO_4_ nor PAA-2H_2_SO_4_ alone produced cellulose nanocrystals, whereas hydrolysis with PAA-10H_2_SO_4_ did generate cellulose nanocrystals which were similar to the ones directly obtained from WC-DFF. The result indicates that the direct extraction of CCNCs by PAA-10H_2_SO_4_ resulted from the cooperative effect of a strong oxidative agent and strong enough acid, which makes the removal of all components, including lignin, hemicellulose, mineral, and wax, occur in parallel with the hydrolysis of amorphous cellulose regions. Notably, the chemical component removal reaction may take place first, followed by the hydrolysis of the cellulose component, but further investigations focusing on, for example, the in situ characterization method and the conditions of chemical reaction, are needed to fully understand the mechanism of the chemical reaction in our method.

Lastly, it is also interesting to compare the efficiency of the current method with others, as shown in Table S1. In the current report, we only draw comparisons with our previous report [56], in which a similar starting material, DFF, was used. The data indicated that the current method used a much lower amount of H_2_SO_4_ (~100 times), and greater amounts of H_2_O_2_ and acetic acid (2.5 times); in addition the current method takes a shorter time (2.5 times) with less electrical power consumer (2.5 times). Our data showed that the current method is more efficient than the conventional method; however, further investigations focusing on, for example, the total cost of CCNC production, environmental impact, and corrosion issues also need to be considered.

## 4. Conclusions

To summarize, we demonstrated an effective, single-step and direct method for directly extracting cellulose nanocrystals from dragon fruit foliage using a mixture of peracetic acid and 10% H_2_SO_4_ solution. We observed that the lignin component, mineral, extractives, and part of the hemicellulose was removed after the treatment. The obtained CCNCs have a high crystalline index (71.3%), with -COOH content of 1.405 mmol/g, which results in a zeta potential value of −48.5 mV. Morphologically, the CCNCs have needled-like shape with a width of 10–50 nm, a length of a few 100 nm (aspect ratio of 2 to 10), and a height of 4 to 10 nm. We attributed the features to the cooperative effect of a strong oxidative agent and a strong enough acid that facilitates the removal of all components occurring simultaneously paralleled with the hydrolysis of amorphous cellulose regions. Our study opens a new, simple method to directly isolate cellulose nanocrystals from lignocellulosic sources, especially dragon fruit foliage. Further research on several lignocellulosic sources is needed to generalize our method as well as determine the effectiveness, environmental impacts, and cost-effectiveness of our method.

## Figures and Tables

**Table 1:** Chemical composition of the raw woody cores of dragon fruit foliage (WC-DFF), the bleached sample (Bleached DFF or Bleached MCC), and the carboxylated cellulose nanocrystals (CCNCs) obtained directly from the reaction between the WC-DFF powder and the PAA-10H_2_SO_4_. Each measurement was performed at least 3 times.

	Materials		
	Raw DFF (%)	Bleached MCC (%)	CCNCs (%)
Cellulose	26.73 ± 4.98	55.94 ± 7.37	67.87 ± 5.20
Lignin	22.05 ± 3.55	1.03 ± 0.57	0.9 ± 0.18
Hemicellulose	28.50 ± 3.15	30.20 ± 4.64	20.60 ± 5.02
Ash	9.55 ± 0.01	0.97 ± 0.32	0.67 ± 0.24
Extractives	7.27 ± 1.01	4.00 ± 5.63	1.10 ± 1.27
Moisture	5.9 ± 1.12	7.86 ± 0.84	8.86 ± 0.38

## Data Availability

The raw data are available from the corresponding author by request.

## Supplementary Materials

The following supporting information can be downloaded at: https://www.mdpi.com/article/10.3390/polym17152124/s1, Figure S1: Thermal analysis of the CCNCs, bleached WC-DFF, and raw samples; Figure S2: Particle distribution size of CCNCs isolated from DFF obtained from DLS measurement; Figure S3: Image and SEM picture of (A) banana pseudostem and cellulose nanocrystals obtained from the reaction between banana pseudostem and PAA-10H_2_SO_4_, and (B) cob corn and cellulose nanocrystals obtained from the reaction between cob corn and PAA-10H_2_SO_4_; Section S1; Table S1: Summary of methods used to extract nanocrystalline cellulose from lignocellulosic source.

## Abbreviations

The following abbreviations are used in this manuscript:

CCNCs	carboxylated cellulose nanocrystals
CNCs	cellulose nanocrystals
WC-DFF	woody cores of dragon fruit foliage
PAA	peracetic acid
FE-SEM	field emission scanning electron microscopy
AFM	atomic force microscopy
MCC	microcrystalline cellulose
