# Supplementary material for: Direct Isolation of Carboxylated Cellulose Nanocrystals from Lignocellulose Source
Source: Polymers (Basel). 2025 Jul 31;17(15):2124. doi: 10.3390/polym17152124 (PMC12349670; doi:10.3390/polym17152124)
Supplement: Supplementary file 1 [file polymers-17-02124-s001.zip › polymers-3653769-supplementary.pdf]

Supplement Information

Direct Isolation of Carboxylated Cellulose Nanocrystals from Lignocellulose Source

Thai Anh Do [1], Luong Lam Nguyen [1], Thuy Khue Nguyen Thi [2] and Van Quyen Nguyen [1,*]

[1] Department of Advanced Materials Science and Nanotechnology, University of Science and Technology of Hanoi, Vietnam Academy of Science and Technology, 18 Hoang Quoc Viet, Cau Giay, Hanoi 11307, Vietnam; anhdt.m22amsn@usth.edu.vn (T.A.D.); nguyen-luong.lam@usth.edu.vn (L.L.N.)

[2] Faculty of Pharmacy, Haiphong University of Medicine and Pharmacy, 72A Nguyen Binh Khiem, Ngo Quyen, Ha Phong 180000, Vietnam; nttkhue@hpmu.edu.vn

* Correspondence: nguyen-van.quyen@usth.edu.vn

Keywords: lignocellulosic source; carboxylated cellulose nanocrystals; dragon fruit foliage; peracetic acid

S1. Quantifying the -COOH content.

The content of the functional group -COOH was quantified using an electrical conductivity method that was adapted from the Canadian Standards Association (CSA), a suggested protocol for the determination of the physical and chemical properties of CNMs entitled Cellulosic Nanomaterials – Test Methods for Characterization (CSA Z5100-14).[1] A freeze-dried sample (0.25 g) was suspended in 100 ml of deionized water. Before titration, 250 µL 0.1 % NaCl (w/v) was added to increase conductivity. Conductimetric titration was performed with 0.1 M NaOH as a titrant. The -COOH content was then quantified using the following equation:

$$-\text{COOH content} = \frac{C(V_1 - V_2)}{W}\ (\text{mmol/g}) \quad \text{(S1)}$$

where V_2 and V_1 are the volumes of 0.1 N NaOH required to neutralize the carboxylic groups, C is the NaOH concentration (mol/L), and W is the freeze-dried sample weight. Each experiment was repeated three times, and the value of the -COOH content was averaged.

S2. *Thermal analysis*

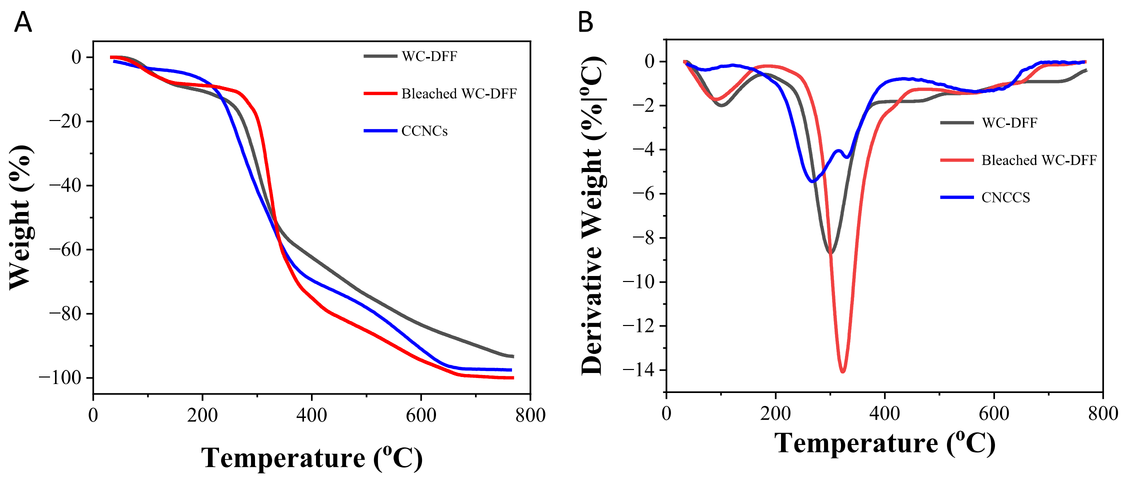

Figure S1. Thermal analysis of the CCNCs, bleached WC-DFF and raw samples.

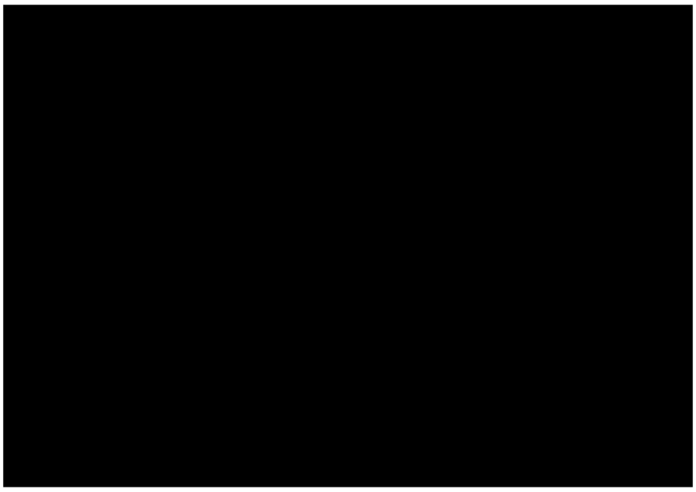

Figure S2. Particle distribution size of CCNCs isolated from DFF obtained from DLS measurement.

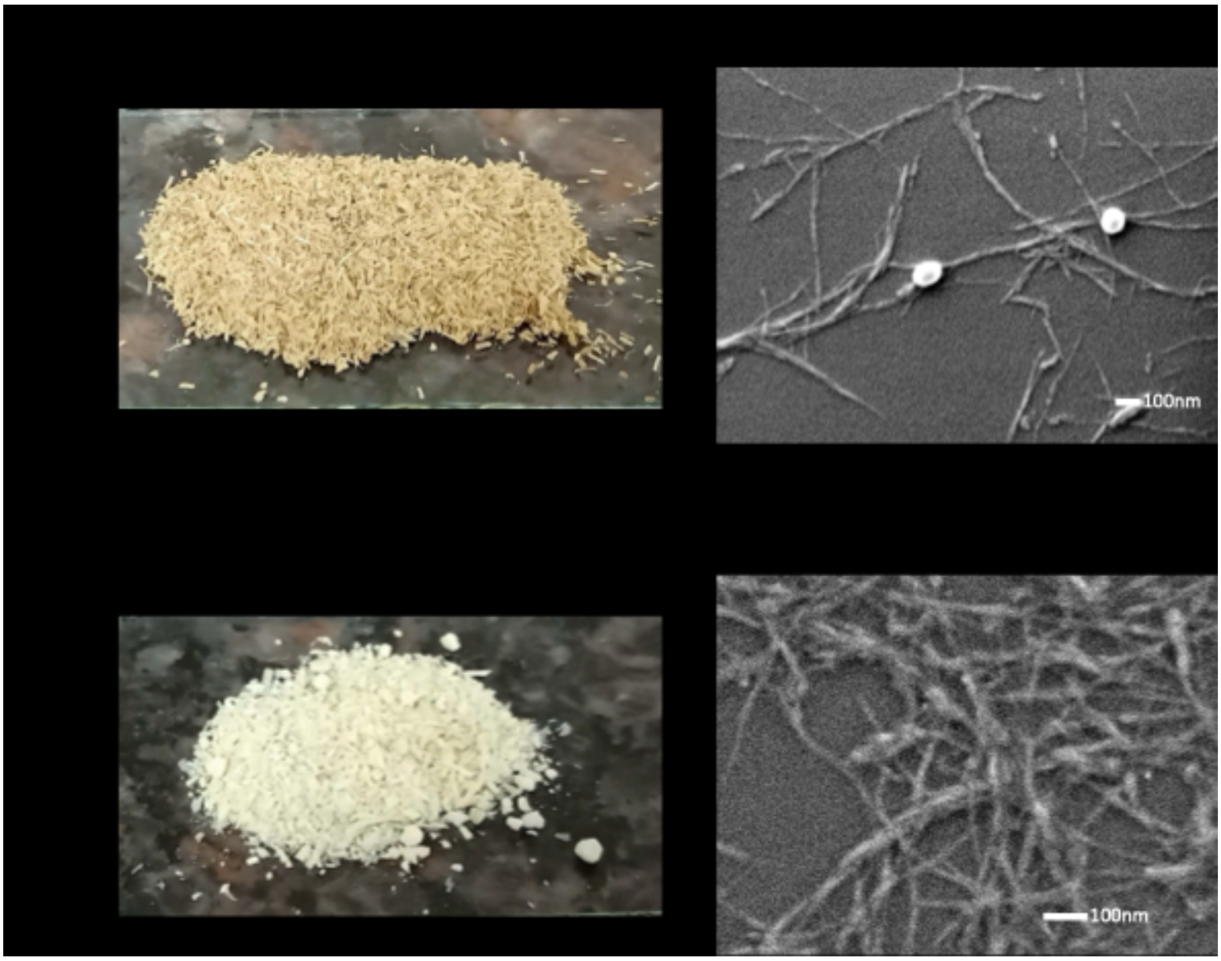

Figure S3. Image and SEM picture of (A) Banana pseudostem and cellulose nanocrystals obtained from the reaction between Banana pseudostem and PAA-10H_2SO_4, and (B) Cob corn and cellulose nanocrystals obtained from the reaction between cob corn and PAA-10H_2SO_4.

Table S1: Summary of methods used to extract nanocrystalline cellulose from lignocellulosic source.

No.	Extraction Method	Lignocellulosic Source	Size (nm)	Ref
1	NaOH → HCl → $NaClO_2$ + CH_3COOH	Water hyacinth	10 - 40	[1]
2	NaOH → H_2O_2 + CH_3COOH → HCl	Water hyacinth	93.02	[2]
3	HCl → NaOH → $NaClO_2$ + CH_3COOH → Mechanical grinding	Pinecone biomass	10 - 25	[3]
4	NaOH → (CH_3COOH + CH_3COONa + $NaClO_2$) × 4 → H_2SO_4 → ultrasonic homogenized	Sugarcane bagasse fibers	5 ± 1.1 × 275 ± 73	[4]

No.	Extraction Method	Lignocellulosic Source	Size (nm)	Ref
5	NaOH → (CH_3COOH + CH_3COONa + $NaClO_2$) × 5 → H_2SO_4 → ultrasonic homogenized	Wood chips from Scots pines and Norway spruce	3.4 ± 0.8 × 162.6 ± 51.8	5
6	NaOH → (CH_3COOH + CH_3COONa + $NaClO_2$) × 5 → H_2SO_4 → ultrasonic homogenized	Branches from Scots pines and Norway spruce	2.8 ± 0.5 × 194.5 ± 64.5	5
7	NaOH → (CH_3COOH + CH_3COONa + $NaClO_2$) × 5 → H_2SO_4 → ultrasonic homogenized	Pine needles from Scots pines and Norway spruce	3.4 ± 0.5 × 166.7 ± 46.5	5
8	Soxhlet extraction → ($NaClO_2$ + CH_3COOH) × 5 → KOH → Homogenization + Ultrasonic	Rice straw	6 - 20	6
9	Alkali treatment → Steam explosion → $NaClO_2$ → Oxalic acid + Sonication	Jute fibers	50	7
10	Ultrasonic-assisted solvent immersion → Alkaline treatment → $NaClO_2$ + CH_3COOH → TEMPO-mediated → Ultrasonication	Coconut husk	5.6 ± 1.5	8
11	Alkali treatment → Steam explosion → $NaClO_2$ → Oxalic acid	Pineapple leaves	5 - 40	9
12	NaOH → Bleaching process → Mechanical grinding	Water retted kenaf bast fibers	1.2 - 34	10
13	$NaClO_2$ + CH_3COOH → NaOH + Urea + Thiourea	Waste paper	50	11
14	Ethanol → NaClO → NaOH → H_2SO_4 → Ultrasonication	Discard cigarette filter	8 × 143	12
15	Ammonium persulfate	Pulp and paper mill	10 – 20 × 150 500	13
16	Steam explosion + Glacial acetic acid + Nitric acid	Wood furniture waste	18 – 40.5	14
17	Kraft cooking process with 23% active akali and 25% sulfidity	Cotton stalk	7.0 – 19.2 × 90.5 – 664.9	15
18	NaOH → Steam explosion → $NaClO_2$ → H_2SO_4	Sisal fibers	60 - 100	16
19	Ball milling + phosphotungstic acid → Ultrasonication	Bamboo pulp	25 – 50 × 200 - 300	17
20	NaOH → Homogenization + Ultrasonication	Pineapple leaf fibers	68 × 88 - 1100	18
21	Steam explosion → HCl → NaOH → Microfluidization treatment	Wheat straw	5.42 × 10 - 40	19

No.	Extraction Method	Lignocellulosic Source	Size (nm)	Ref
22	Microwave-assisted delignification + $NaClO_2$ → Ultrasound + NaOH → H_2SO_4	Corn cob	131.4	20
23	NaOH → H_2O_2 → Ammonium persulphate	Rice straw	19 ± 5 × 118 ± 52	21
24	NaOH → H_2SO_4	Garlic straw residues	6 × 480	22
25	NaOH → H_2SO_4	Coffee husk	20 ± 4 × 310 ± 160	23

References

1. Asrofi, M., Abral, H., Kasim, A. & Pratoto, A. XRD and FTIR Studies of Nanocrystalline Cellulose from Water Hyacinth (<i>Eichornia crassipes</i>) Fiber. *Journal of Metastable and Nanocrystalline Materials* 29, 9–16 (2017).

2. Packiam, K. K., Murugesan, B., Kaliyannan Sundaramoorthy, P. M., Srinivasan, H. & Dhanasekaran, K. Extraction, Purification and Characterization of Nanocrystalline Cellulose from *Eichhornia crassipes* (Mart.) Solms: A Common Aquatic Weed Water Hyacinth. *Journal of Natural Fibers* 19, 7424–7435 (2022).

3. Rambabu, N., Panthapulakkal, S., Sain, M. & Dalai, A. K. Production of nanocellulose fibers from pinecone biomass: Evaluation and optimization of chemical and mechanical treatment conditions on mechanical properties of nanocellulose films. *Ind Crops Prod* 83, 746–754 (2016).

4. El Miri, N. *et al.* Synergistic effect of cellulose nanocrystals/graphene oxide nanosheets as functional hybrid nanofiller for enhancing properties of PVA nanocomposites. *Carbohydr Polym* 137, 239–248 (2016).

5. Moriana, R., Vilaplana, F. & Ek, M. Cellulose Nanocrystals from Forest Residues as Reinforcing Agents for Composites: A Study from Macro- to Nano-Dimensions. *Carbohydr Polym* 139, 139–149 (2016).

6. Dilamian, M. & Noroozi, B. A combined homogenization-high intensity ultrasonication process for individualizaion of cellulose micro-nano fibers from rice straw. *Cellulose* 26, 5831–5849 (2019).

7. Thomas, M. G. *et al.* Nanocelluloses from jute fibers and their nanocomposites with natural rubber: Preparation and characterization. *Int J Biol Macromol* 81, 768–777 (2015).

8. Wu, J. *et al.* Preparation and characterization of cellulose nanofibrils from coconut coir fibers and their reinforcements in biodegradable composite films. *Carbohydr Polym* 211, 49–56 (2019).

9. Abraham, E. *et al.* Extraction of nanocellulose fibrils from lignocellulosic fibres: A novel approach. *Carbohydr Polym* 86, 1468–1475 (2011).

10. Karimi, S., Tahir, P. Md., Karimi, A., Dufresne, A. & Abdulkhani, A. Kenaf bast cellulosic fibers hierarchy: A comprehensive approach from micro to nano. *Carbohydr Polym* 101, 878–885 (2014).

11. Zhang, S. *et al.* Preparation of spherical nanocellulose from waste paper by aqueous NaOH/thiourea. *Cellulose* 26, 5177–5185 (2019).

12. Ogundare, S. A., Moodley, V. & van Zyl, W. E. Nanocrystalline cellulose isolated from discarded cigarette filters. *Carbohydr Polym* 175, 273–281 (2017).

13. Gibril, M. E. *et al.* Beneficiation of pulp and paper mill sludge: production and characterisation of functionalised crystalline nanocellulose. *Clean Technol Environ Policy* 20, 1835–1845 (2018).

14. de Oliveira, P. B., Godinho, M. & Zattera, A. J. Oils sorption on hydrophobic nanocellulose aerogel obtained from the wood furniture industry waste. *Cellulose* 25, 3105–3119 (2018).

15. Li, M., He, B., Chen, Y. & Zhao, L. Physicochemical Properties of Nanocellulose Isolated from Cotton Stalk Waste. *ACS Omega* 6, 25162–25169 (2021).

16. Vishnoi, Y. *et al.* Extraction of nano-crystalline cellulose for development of aerogel: Structural, morphological and antibacterial analysis. *Heliyon* 10, e23846 (2024).

17. Lu, Q. *et al.* Extraction of Cellulose Nanocrystals with a High Yield of 88% by Simultaneous Mechanochemical Activation and Phosphotungstic Acid Hydrolysis. *ACS Sustain Chem Eng* 4, 2165–2172 (2016).

18. Mahardika, M., Abral, H., Kasim, A., Arief, S. & Asrofi, M. Production of Nanocellulose from Pineapple Leaf Fibers via High-Shear Homogenization and Ultrasonication. *Fibers* 6, 28 (2018).

19. Liu, Q. *et al.* Isolation of High-Purity Cellulose Nanofibers from Wheat Straw through the Combined Environmentally Friendly Methods of Steam Explosion, Microwave-Assisted Hydrolysis, and Microfluidization. *ACS Sustain Chem Eng* 5, 6183–6191 (2017).

20. Louis, A. C. F. & Venkatachalam, S. Energy efficient process for valorization of corn cob as a source for nanocrystalline cellulose and hemicellulose production. *Int J Biol Macromol* 163, 260–269 (2020).

21. Oun, A. A. & Rhim, J.-W. Isolation of oxidized nanocellulose from rice straw using the ammonium persulfate method. *Cellulose* 25, 2143–2149 (2018).

22. Kallel, F. *et al.* Isolation and structural characterization of cellulose nanocrystals extracted from garlic straw residues. *Ind Crops Prod* 87, 287–296 (2016).

23. Collazo-Bigliardi, S., Ortega-Toro, R. & Chiralt Boix, A. Isolation and characterisation of microcrystalline cellulose and cellulose nanocrystals from coffee husk and comparative study with rice husk. *Carbohydr Polym* 191, 205–215 (2018).
